# Monitoring in practice – How are UK academic clinical trials monitored? A survey

**DOI:** 10.1186/s13063-019-3976-1

**Published:** 2020-01-09

**Authors:** Sharon B. Love, Victoria Yorke-Edwards, Sarah Lensen, Matthew R. Sydes

**Affiliations:** MRC Clinical Trials Unit at UCL, 90 High Holborn, London, WC1V 6LJ UK

**Keywords:** Clinical trial monitoring, On-site monitoring, Central monitoring, Trial conduct, Remote monitoring

## Abstract

**Background:**

Despite the US Food and Drug Administration (FDA) and European Medicines Agency (EMA) encouraging the use of risk-based monitoring for trials in 2013, there remains a lack of evidence-based guidelines on how to monitor. We surveyed the academic United Kingdom Clinical Research Collaboration (UKCRC) registered clinical trials units (CTUs) to find out their policy on monitoring of phase III randomised clinical trials of an investigational medicinal product (CTIMPs).

**Methods:**

An online survey of monitoring policy with sections on the CTU, central monitoring and on-site monitoring was sent to all 50 UKCRC registered CTUs in November 2018.

Descriptive data analysis and tabulations are reported using the total number answering each question.

**Results:**

A total of 43/50 (86%) of CTUs responded with 38 conducting phase III randomised CTIMP trials. Of these 38 CTUs, 34 finished the survey. Most CTUs (36/37, 97%) use a central monitoring process to guide, target or supplement site visits. More than half (19/36, 53%) of CTUs do not use an automated monitoring report when centrally monitoring trials and all units use trial team knowledge to make a final decision on whether an on-site visit is required.

A total of 31/34 (91%) CTUs used triggers to decide whether or not to conduct an on-site monitoring visit. On-site, a mixture of source data verification and checking of processes was carried out.

The CTUs overwhelmingly (27/34, 79%) selected optimising central monitoring as their most pressing concern.

**Conclusion:**

The survey showed a wide variation in phase III randomised CTIMP trial monitoring practices by academic clinical trials units within a single research-active country. We urgently need to develop evidence-based regulator-agreed guidance for CTUs on best practice for both central and on-site monitoring and to develop tools for all CTUs to use.

## Background

Clinical trialists monitor trial data in order to protect the rights and well-being of participants, to ensure that the trial data are accurate, complete, and verifiable, and to confirm that the trial is being run in compliance with the currently approved protocol, with the principles of good clinical practice (GCP), and with the relevant regulatory requirements [1]. In 2013, publications from both the US Food and Drug Administration (FDA) [2] and European Medicines Agency (EMA) [3] promoted trial sponsors moving to a risk-based approach to monitoring. In risk-based monitoring, the monitoring activities are directed at preventing or mitigating important and likely risks to data quality, to processes critical to human subject protection and to trial integrity. Rather than monitoring routinely throughout the trial, the monitoring is directed at any risks to the trial. The new interest in risk-based monitoring was subsequently enshrined in the International Council for Harmonisation of Technical Requirements for Pharmaceuticals for Human Use (ICH) GCP E6(R2) [1] guidance in December 2016. This publication gave the advice:

*The sponsor should develop a systematic, prioritized, risk-based approach to monitoring clinical trials. […] The sponsor may choose on-site monitoring, a combination of on-site and centralized monitoring, or, where justified, centralized monitoring.*

The monitoring literature of interventional trials and studies is surprisingly sparse. Three Studies Within A Trial (SWATs) have been published in this area, showing that compared to full monitoring, risk-based monitoring is a reasonable approach [4–6]. Tudur Smith et al. [7] and Embleton-Thirsk et al. [8] have compared the analyses of the clinical trial data collected centrally by clinical trials units (CTUs) with that collected by using 100% on-site source data verification. Each found that the extra on-site monitoring made little difference to the primary efficacy results of the trial, and these results were their key dissemination message. The TEMPER study [9] showed that triggers based on centrally stored data are potentially a good way of being able to choose which sites of a clinical trial to visit providing there are no consent issues. Most recently, the START monitoring substudy published their results [10] showing the benefit and cost of on-site monitoring. Balancing these two issues, they concluded that the value to the START trial of onsite monitoring was limited.

In the last 10 years, three surveys have reported how trialists deal with certain aspects of monitoring. Morrison [11] reported a survey with a 37% (79/216) response rate carried out in late 2009 of pharmaceutical, regulatory and academic groups in the USA conducting clinical trials. From a maximum of 65 respondents they found that 78% (46/59) always or sometimes used risk-based monitoring, 83% (48/58) used central data to evaluate site performance and that 70% (42/60) always did site visits. Tudur Smith [12] reported a 2011 monitoring survey with a 48% (23/48) response rate of the then 48 United Kingdom Clinical Research Collaboration (UKCRC) registered CTUs. These are UK academic CTUs that have been assessed as reaching a standard by an international panel of experts in clinical trials research. Risk-based monitoring was being used by 53% of CTUs (number not given, maximum number of respondents 22) and most CTUs used some level of central monitoring for each trial, sometimes supplemented by on-site monitoring. Beever and Swaby [13] reported a 2017 survey of the then 49 UKCRC registered CTUs on the assessment of risk in risk-based monitoring. All 23 respondents (response rate 47% (23/49)) carried out risk-based monitoring with 96% (22/23) using a combination of remote and on-site monitoring.

There is clearly an appetite for risk-based monitoring among regulators, which has been supported by the studies assessing this approach. However, there are few evidence-based guidelines on best practice and no recent data on how monitoring post risk assessment is currently performed. To determine current monitoring practice, we sent a survey to the 50 UKCRC registered CTUs to determine their policy on monitoring phase III randomised CTIMP (Clinical Trial of an Investigational Medicinal Product) trials.

## Methods

We aimed to find out each UKCRC registered CTU’s policy on monitoring their phase III randomised CTIMP trials. The survey included questions on central and on-site monitoring, considering both data quality and whether the trial was being run appropriately at each site. We defined central monitoring as monitoring using data collected at the CTU and on-site monitoring as monitoring where a visit is made to the site where the source data is collected.

### Survey development

There was no pre-existing robust tool for collecting this information. We created our survey by adapting the survey questions of Morrison [11] to the UK registered CTU situation. We asked (i) four questions to determine the typical characteristics of the phase III randomised CTIMP trials that the CTU carried out, (ii) eight questions about central (within CTU) monitoring and (iii) nine questions about on-site monitoring. We gave space for comments in each of the three sections. Screenshots of the survey are provided in Additional file 1 and the questionnaire transcript is given in Additional file 2. The survey’s content validity was checked by all authors. The survey was piloted by eight clinical project managers at the MRC Clinical Trials Unit at UCL (MRC CTU at UCL) to check content and ensure ease of completion. The authors agreed upon the final question selection and wording.

Definitions for ‘central monitoring’ and ‘triggers’ were included in the survey to ensure consistency. Central monitoring was defined as any monitoring of the data or the sites that the CTU covers from the trials office, but it excluded the automatic queries programmed in the database used during data entry. This broad definition was used to cover central statistical monitoring, trial managers looking at reports and fully programmed triggering systems delivering the names of sites requiring a visit. Triggers were defined as the means by which CTUs decide to visit a site based on the information held centrally in the CTU.

The survey was set up in Opinio [14] enabling online completion. Care was taken to make the questions easy to read and complete, with screens of questions ordered into sections and notification given on each screen of how many questions had been answered out of the total.

### Eligibility and selection

We sent the survey to the then 50 UKCRC registered CTUs in the UK because they have reached a demonstrable standard (registration criteria) and therefore should reflect current best practice within a single research-active country. UK CTUs are specialised units within universities, hospitals or institutes who design, conduct, analyse and publish many multicentre trials. They have expertise in trial management, statistics and trial design, and often have medical experts; this core team of experts ensures trials are conducted to meet appropriate standards and regulations. Chief investigators may be part of the CTU or external to it. Staff and trial funding is often from a mixture of charity and university funding with peer-reviewed grants achieved from charity, government and industry funders for each trial [15]. Fully registered CTUs have a proven track record with robust quality assurance systems, evidence of long-term viability of trial coordination, and a trials portfolio; provisionally registered CTUs are those that do not meet the criteria of full registration but plan to within 3 years. Monitoring practice varies across the trial phases with more intensive monitoring in phase I trials, so we limited the survey to CTUs that conduct phase III trials. The conduct of CTIMPs is inspected by the Medicines and Healthcare products Regulatory Agency (MHRA) as they are considered to have higher associated risks than non-CTIMPs. Randomised trials are considered to be the gold standard of interventional research; we therefore chose to further limit the survey to CTUs that run randomised CTIMPs.

### Survey distribution

The survey link was emailed by the Senior Administrative Assistant of the UKCRC Registered CTU Network on 13 November 2018 to each CTU Director, asking them to pass it to the monitoring lead in the unit. A reminder email was sent by the Senior Administrative Assistant to all CTU Directors on 3 December 2018. If a CTU had still not completed the survey, an alternative contact at the CTU was sent a personal message from an author (SBL) on 11 December 2018, with a final reminder email on 17 December 2018. The final response was received on 20 December 2018.

### Ethics and consent

We did not require ethics permission as invitees were non-NHS staff and we inferred implied consent when we received a response. The aims of the survey were described in the email and on the first screen of the survey. We collected each CTU’s name in order to avoid duplication and to facilitate reminders but we agreed not to use this in the analysis or dissemination.

### Analysis

The raw data was downloaded from Opinio and stored as per MRC CTU at UCL policy, with only those involved in the survey having access. Descriptive analyses were performed using Stata version 15.1 [16] including all responses on each question.

We attempted to identify differences between the group of CTUs that responded and those that did not through the information on the UKCRC registered CTU website [17]. This information included whether they conduct cancer trials, conduct international trials, carry out 24-hour randomisation, or have full or provisional UKCRC registration.

## Results

The majority of UKCRC registered CTUs responded to the survey (86% (43/50)), including five who clarified that they do not run phase III CTIMP trials. CTUs that do not carry out 24-hour randomisation were less likely to respond to the survey (Table 1).

**Table 1:** A comparison of invited CTUs response status and four key characteristics

CTU characteristics	Answered survey N (%)	Did not answer survey N (%)	Chi square and *p* value for answering survey comparison	Those eligible for survey N (%)
Registration status				
Full	39 (91)	6 (86)	0.2	36 (95)
Provisional	4 (9)	1 (14)	*p* = 0.7	2 (5)
Cancer trials				
Yes	30 (70)	4 (57)	0.4	28 (74)
No	13 (30)	3 (43)	*p* = 0.5	10 (26)
24-hour randomisation				
Yes	38 (88)	4 (57)	4.4	33 (87)
No	5 (12)	3 (43)	*p* = 0.04	5 (13)
International trials				
Yes	29 (67)	6 (86)	1.0	25 (66)
No	14 (33)	1 (14)	*p* = 0.3	13 (34)

*CTU* clinical trials unit

A maximum of 38 CTUs completed at least one survey question and their characteristics are described in the last column of Table 1. The questionnaire took a median 19 minutes to complete, interquartile range (8.5, 64.0). As some CTUs did not answer every question, the actual number doing so for each question is given. Many CTUs considered their typical phase III randomised CTIMP trial to have 101–1000 patients and 11–49 sites (Table 2).

**Table 2:** Number of participants and sites for phase III randomised CTIMP trials run by included CTUs

Number of sites	Number of patients					Total
	1–100	101–1000	1001–2499	2500+	No answer given	
1–10	1	4	0	0	0	5
11–49	2	14	5	0	1	22
50+	0	5	3	3	0	11
Total	3	23	8	3	1	38

*CTIMP* clinical trial of an investigational medicinal product, *CTU* clinical trials unit

A total of 28/38 (74%) of CTUs had some non-UK sites. For all CTUs an assessment of risk informed their monitoring approach, although for one CTU this was only sometimes and for four CTUs this assessment of risk was done by the sponsor.

For one CTU, the sponsor had responsibility for all monitoring, so they could not complete the sections on central and on-site monitoring. Thirty-four of the remaining 37 CTUs completed the questionnaire.

### Central monitoring

Almost all CTUs use centrally available data to evaluate site performance (34/37, 92%) with two further CTUs (total 36/37, 97%) using a central monitoring process to guide, target or supplement site visits. One sixth (6/36, 17%) reported never using a centralised monitoring process to replace on-site visits, while two reported always doing so (2/36, 6%).

Over half reported running central monitoring processes at least once per month on each trial (20/35, 57%) with only one (3%) running them just annually.

For more than half of CTUs (19/36, 53%), central monitoring is not explicitly programmed, i.e. standard reports may be used, but a monitoring report is not automatically produced. For the remainder, 5 (14%) use the same monitoring programming code for all of their trials, 4 (11%) choose pre-written modules with some bespoke programming and 8 (22%) write bespoke software programming for each trial.

**Fig. 1:**
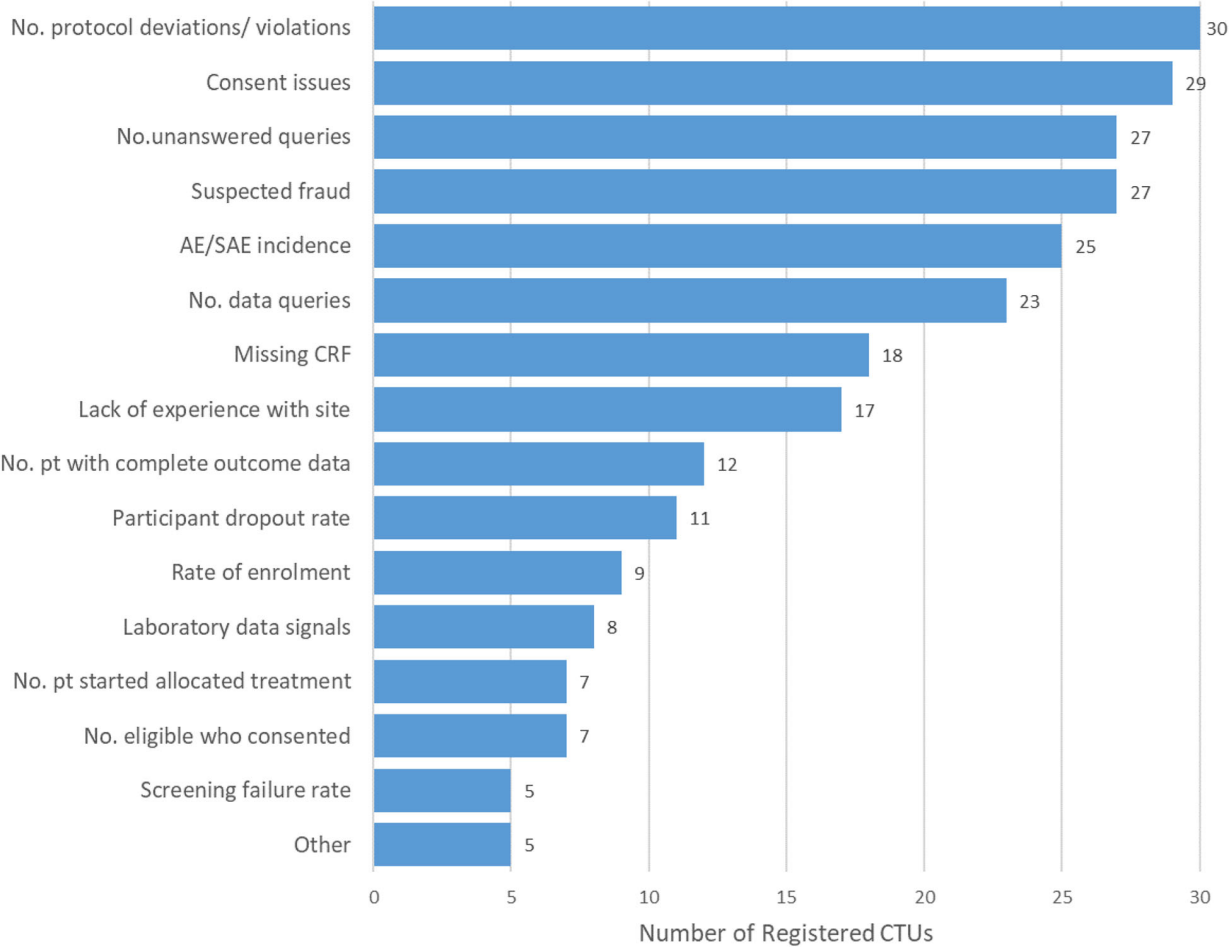
Frequency of factors likely to trigger an on-site monitoring visit. CTU could choose multiple options. *CTU* clinical trial unit, *No* number, *pt* participant

The assessment of triggers showing a site should be visited is not solely defined by software for any CTU, instead there is always human input in choosing which sites to visit. Figure 1 show the factors likely to trigger an on-site monitoring visit.

### On-site monitoring

All 34 CTUs responding to the on-site questions performed on-site monitoring at least sometimes, with most CTUs finding the on-site visit to take 1 day (27/34, 79%) and needing one person (27/34, 79%). This person was often a trial manager or dedicated monitor (18/34, 53%) but in some cases could be a member of the CTU’s Quality Assurance team, the Chief Investigator or a member of staff from a contract research organisation (CRO) (Additional file 3).

Most CTUs used formal triggers to decide whether or not to conduct an on-site monitoring visit (31/34, 91%). Of these, only one (1/31, 3%) solely used triggers to choose whether to conduct an on-site visit, with the remainder also conducting some on-site visits after fixed time periods, based on the number of patients that had been recruited, because of a trial event (e.g. independent data monitoring committee [IDMC]), or for a mixture of these reasons.

**Fig. 2:**
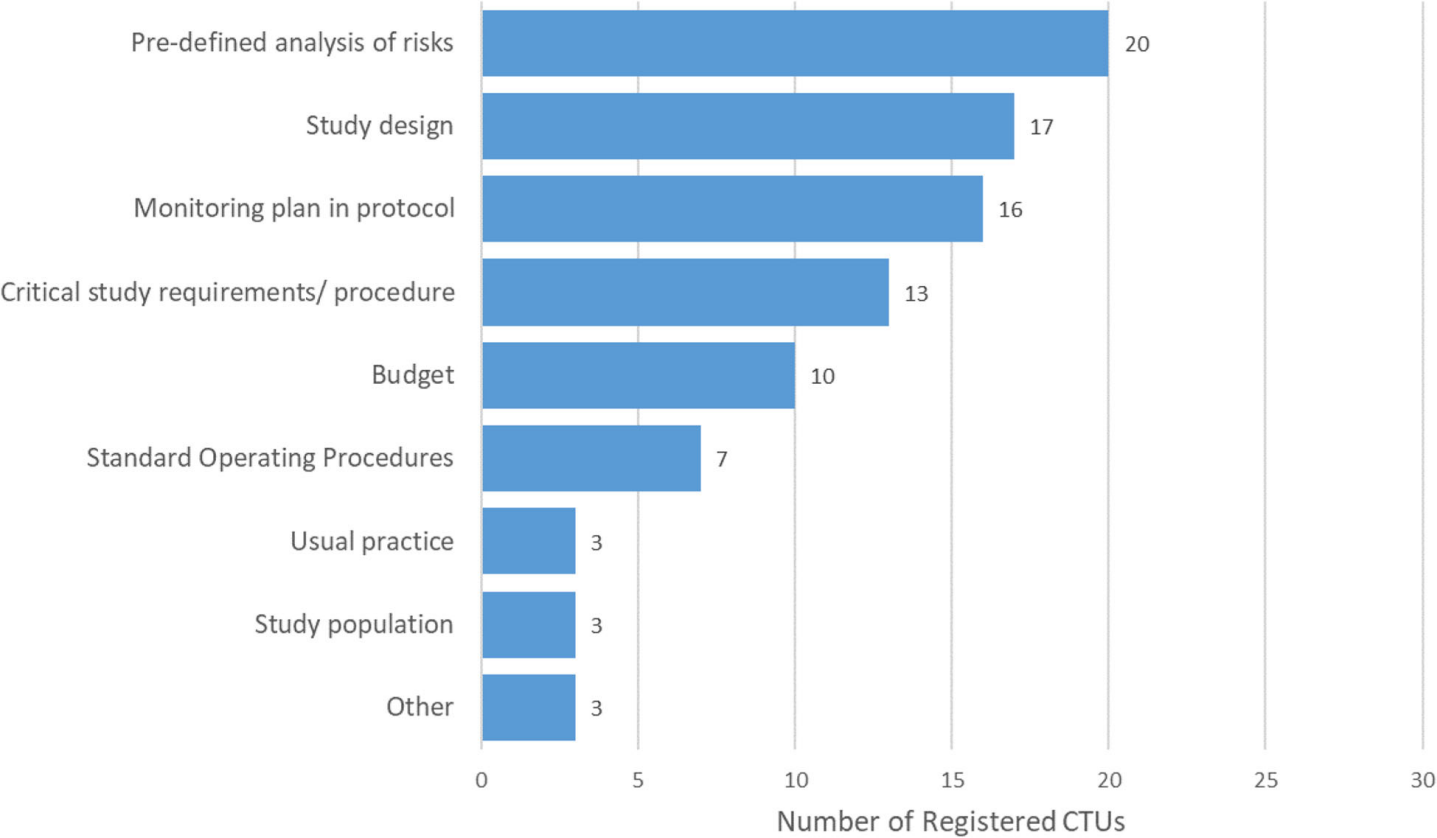
Reasons for frequency of on-site monitoring visits. CTU could choose multiple options. *CTU* clinical trial unit

The stated reasons behind the frequency of on-site visits are given in Fig. 2.

The pre-defined analysis of risks, the study design and the monitoring plan were each listed by more than 20/34 (59%) of CTUs as being a reason behind the frequency of on-site visits. We asked how much on-site source data verification (SDV) was done for various classifications of data. Eight CTUs commented that the question was too difficult to answer as a unit policy across all their trials due to the variability of SDV even within phase III randomised CTIMP trials and so this question was attempted by 24 CTUs (Table 3). One CTU (1/24, 4%) never did any SDV.

**Table 3:** Percentage of SDV done for differing classifications of data

	%SDV									Total
	100	60	50	30	20	15	10	5	0	
All data	0	0	2	1	3	1	5	1	2	15
Consent	**19**	0	2	1	0	0	0	0	0	22
Eligibility criteria	**13**	0	2	1	0	0	1	1	1	19
Primary endpoint reports	**13**	0	0	1	0	0	3	1	0	18
Secondary endpoint reports	4	1	1	1	1	0	6	1	2	17
SAE: Serious adverse event reports	**14**	0	1	1	0	0	1	1	1	19
AE: Non-serious adverse event reports	4	0	0	1	1	0	7	1	3	17
Selected priority data	8	0	0	1	0	0	3	1	5	18

Bold font shows where there appears to be a consensus, i.e. where more than two thirds of the respondents gave the same answer. Columns represent the %SDV that CTUs gave in response to the question

*AE* adverse event, *CTU* clinical trial unit, *SAE* serious adverse event, *SDV* source data verification

Many CTUs reported doing 100% SDV of consent, eligibility criteria, primary endpoint reports and serious adverse events at any given visit.

Other activities achieved during on-site visits are detailed in Table 4.

**Table 4:** Other activities achieved during on-site visits

	Always	Frequently	Occasionally	Never	N/A	Not sure	Total
Assess staff’s understanding of study procedures	**12**	**14**	1	0	0	0	27
Assess the ability of staff to explain study to participants	4	4	12	10	0	2	32
Assess the adequacy and timelines of additional information provided to participants	3	5	15	7	1	1	32
Assess informed consent updates/modifications	**20**	**9**	1	1	1	0	32
Assess regulatory documents and communications	**12**	**13**	6	0	2	0	33
Assess the security of study data and documentation	10	11	9	2	0	1	33
Check the site file is complete	**15**	**16**	2	0	0	0	33
Check adherence to GDPR	7	11	7	6	0	1	32

Bold font shows where more than two thirds of the respondents always or frequently did an activity

*GDPR* General Data Protection Regulation, *N/A* not applicable

Many CTUs frequently or always assessed the site staff’s understanding of study procedures (96%, 26/27), informed consent updates/modifications (91%, 29/32), regulatory documents and communications (76%, 25/33) and checked the site file was complete (94%, 31/33).

### Other results

Table 5 shows what aspect of monitoring the CTUs would most like to change. We gave the top three options plus an ‘other’ category. The majority of CTUs would most like to optimise central monitoring (27/34, 79%).

**Table 5:** Aspects of monitoring the CTU would most like to change

Frequency	Aspect of monitoring CTU would most like to change
27	Optimise central monitoring
5	Stop or reduce SDV
1	Stop or reduce the number of on-site visits
1	Other - have funding for more on-site monitoring visits
34	Total

*CTU* clinical trial unit, *SDV* source data verification

## Discussion

Our survey collected valuable information on how the UKCRC registered CTUs monitor phase III randomised CTIMP trials. The main finding is the wide variety of ways in which central and on-site monitoring are conducted. The survey showed for central monitoring a variety of use, method, frequency of execution, method of trigger assessment and items in triggers. For on-site monitoring, variety was shown in who attended, how many attended, how long site visits were, how to decide when to visit a site, the determinants of a site visit and what to do when there, in terms of SDV and monitoring processes.

Although almost all responding CTUs (36/37 97%) used central monitoring to guide, target or supplement on-site visits and more than half of these CTUs run central monitoring at least monthly (20/36, 56%), for many (19/36, 53%) the central monitoring is not programmed (fully automated). This would likely form a large and repeated burden on the trial management team, adding to the cost of running the trial. It should be possible to fully program this work, with the programming overhead being less than the trial management team burden for longer trials.

Our survey showed that a variety of people are involved in the on-site monitoring. Though this is related in part to the differing job titles used in the CTUs, it would be good to know if the CTUs using specific monitors (19/34, 56%) found there was an advantage in this practice.

The variety of items triggering on-site monitoring and their varying frequency of use shows that there is considerable scope for prospective research to better specify where CTUs should expend their energy. Whitham et al. [18] suggest eight ‘performance metrics’ to be used for all trials alongside just one or two trial-specific metrics. TransCelerate has published eight overlapping metrics [19]. These suggested metrics need prospective testing and reporting of experiential data to show whether they work.

Our survey showed that many CTUs claim to do 100% SDV of some data. However, the two publications looking at this has shown 100% SDV does not change the primary efficacy trial results [7, 8]. SDV may be necessary if particular data have been assessed as being critical to the trial and only able to be monitored in this way, but the FDA has set out guidelines [2] encouraging trialists to consider the needs of each specific trial rather than assume that all trials need SDV.

The Morrison survey from 2009 [11] acquired data from 65 mainly US groups performing trials and concluded that there was a wide variety of monitoring practices and called for research to develop an evidence base for monitoring practice. Our UK survey, nearly a decade later reaches the same conclusions. The previous surveys of UKCRC registered CTUs in 2011 [12] and 2017 [13] agreed with the current survey, also finding that all CTUs at least sometimes informed their monitoring plan through a risk assessment and that most CTUs used some level of central monitoring, either with or without on-site monitoring. Our survey gives more information on how the monitoring is actually done.

There are several limitations to our study. Although the response rate of 86% was high (and double that of the other monitoring surveys), there was complete data from only 34 CTUs. This may be considered a small number to represent the monitoring processes of UK clinical trials. It is limited to the UKCRC registered CTUs who have demonstrated a sufficiently high standard to achieve UKCRC accreditation. These are not fully representative of academic clinical trials units in the UK who are not registered, though fewer randomised phase III CTIMP trials are run outside registered CTUs. The monitoring set-up of academic trials units in other countries and of industry-led or contract research organisation-coordinated trials may differ. However, the information here should be useful to all groups running trials of any phase.

As survey data of policy across a CTU, this information on monitoring lacks detail and does not represent the monitoring that happens on a specific trial. This is more obvious on the SDV questions where eight CTUs felt the SDV was so variable they could not complete the table. It may be that our results are of an ideal world situation.

The findings of this survey have highlighted the need for clarity with regard to terminology in monitoring; multiple definitions for single terms are problematic when communicating with practitioners and researchers across the field of clinical trials. For example, in our survey we used the word ‘triggers’ to describe the items that are reviewed to decide whether a site visit is needed. However, trialists and researchers use differing terms such as triggers, metrics and possibly other terms that our team has not yet come across. On this point, we favour using the term ‘metrics’ and considering visiting a site if the metric threshold is breached. As a community, we need to show how the quality tolerance limits mentioned by ICH [1] relate to metrics. There is also confusion between data cleaning and monitoring, particularly since a monitoring outcome is to prompt for data corrections/clarification. Clarifying the terminology will go a long way to enabling us to move this area forward.

Some research on whether monitoring is best carried out by dedicated monitors, both centrally and on-site would be useful. Our terminology of jobs and some idea of the job description part of whoever is tasked with the monitoring would be beneficial.

With the strong steer from the surveyed CTUs, the next priority is optimising the processes of central monitoring. We think the metrics that are used to improve the data integrity and patient safety need research to confirm that they are helpful, to select which are required, to clarify how they should be used (particularly in terms of frequency and post-metric actions) and to consider where they sit (are they a way of improving the data integrity and the patients safety or are they a way of judging it?).

We think there is research work to be conducted on how on-site visits are best performed. Should we be looking at SDV, processes or a mixture of these? And are site visits necessary or could the effect of a site visit be replicated in other ways by staying at the CTU?

We would like monitoring to get to the same place as protocols and statistical analysis plans for clinical trials have reached (Spirit guidelines [20], Statistical analysis plan guidelines [21],). As part of their systematic approach to making trials more efficient by improving all trial processes, Trial Forge [22] is aiming to increase the evidence base for efficient trial monitoring. We see a practical monitoring guidance document that shows, for differing risks of trials and individual processes, the monitoring that is required. This could perhaps be in the form of a template data monitoring plan.

## Conclusions

This survey provides valuable policy information on how academic CTUs are conducting monitoring in phase III randomised CTIMP trials. All of the responding UKCRC registered CTUs carried out a risk assessment to inform their monitoring approach, the majority did central monitoring and all did on-site monitoring. This monitoring was carried out in a variety of ways. The CTUs have resoundingly called for optimising the central monitoring as the feature of monitoring they would most like to change. We urgently need to give evidence-based regulator-agreed guidance to CTUs on best practice for both central and on-site monitoring and to develop tools for all CTUs to use.

## Supplementary information

**Additional file 1.** Screenshot CRCUK registered CTU questionnaire.

**Additional file 2.** Transcript CRCUK registered CTU questionnaire.

**Additional file 3.** Frequency of differing combinations of people attending on on-site monitoring visits.

## Data Availability

The dataset supporting the conclusions of this article and a copy of the questionnaire text is available in the UCL Research Data Repository, 10.5522/04/7992998.

## Abbreviations

CRO: Contract Research Organisation
CTIMP: Clinical Trial of an Investigational Medicinal Product
CTU: Clinical Trials Unit
EMA: European Medicines Agency
FDA: US Food and Drug Administration
GCP: Good Clinical Practice
ICH: International Council for Harmonisation of Technical Requirements for Pharmaceuticals for Human Use
IDMC: Independent data monitoring committee
MHRA: Medicines and Healthcare products Regulatory Agency
MRC CTU at UCL: MRC Clinical Trials Unit at UCL
SDV: Source data verification
SWAT: Study Within A Trial
UKCRC Registered CTU Network: United Kingdom Clinical Research Collaboration Registered Clinical Trials Unit Network
