# Supplementary figures and images for: Monitoring in practice – How are UK academic clinical trials monitored? A survey
Source: Trials. 2020 Jan 9;21:59. doi: 10.1186/s13063-019-3976-1 (PMC6953230; doi:10.1186/s13063-019-3976-1)

CRCUK registered Clinical Trial Units Questionnaire


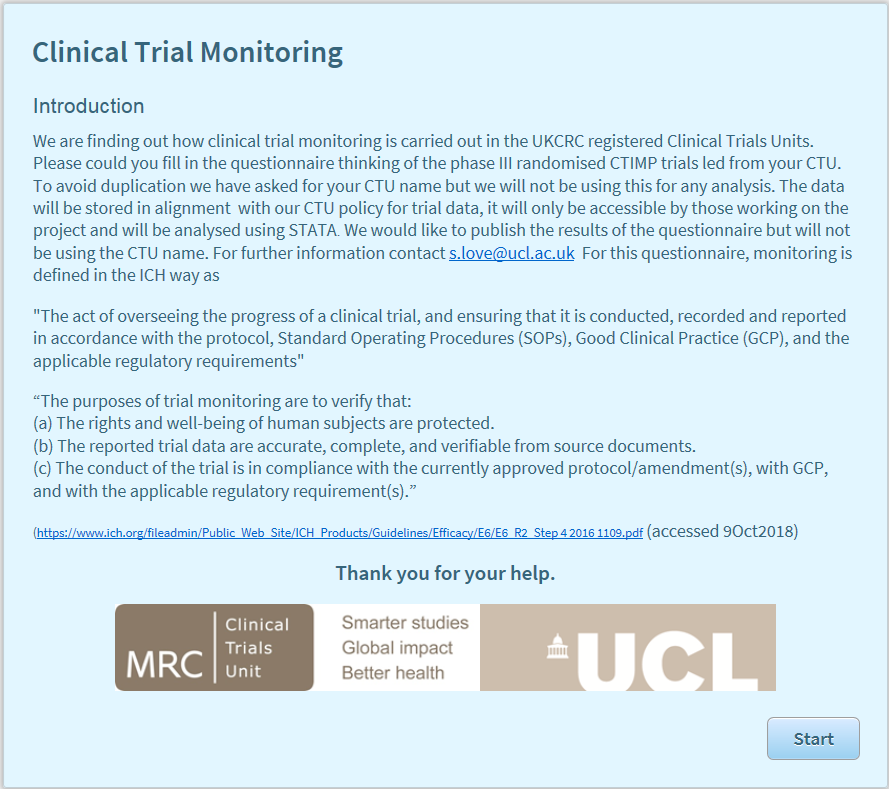


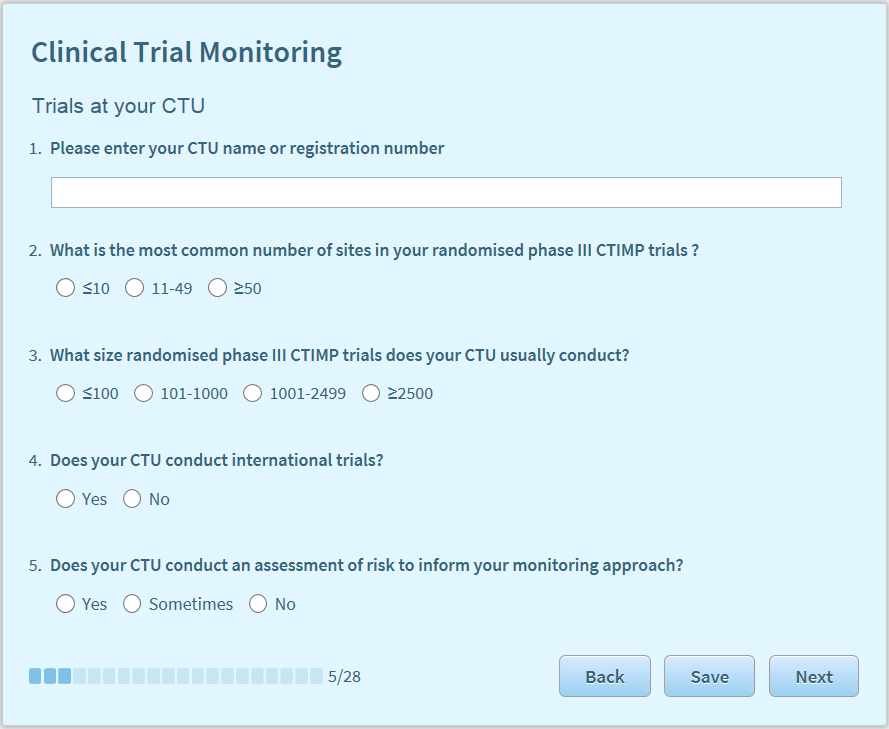


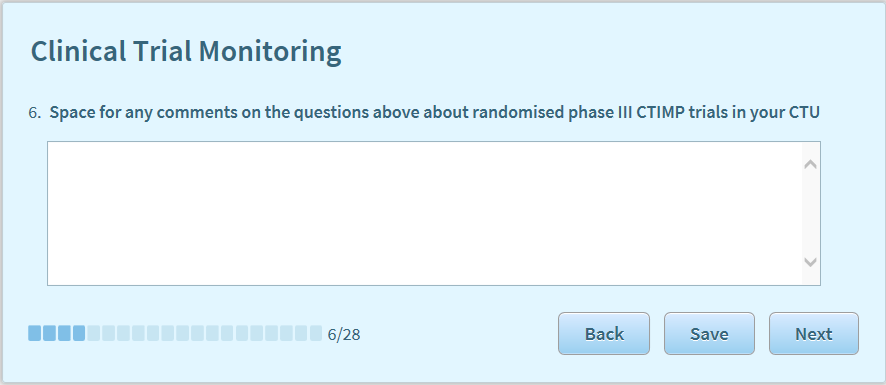


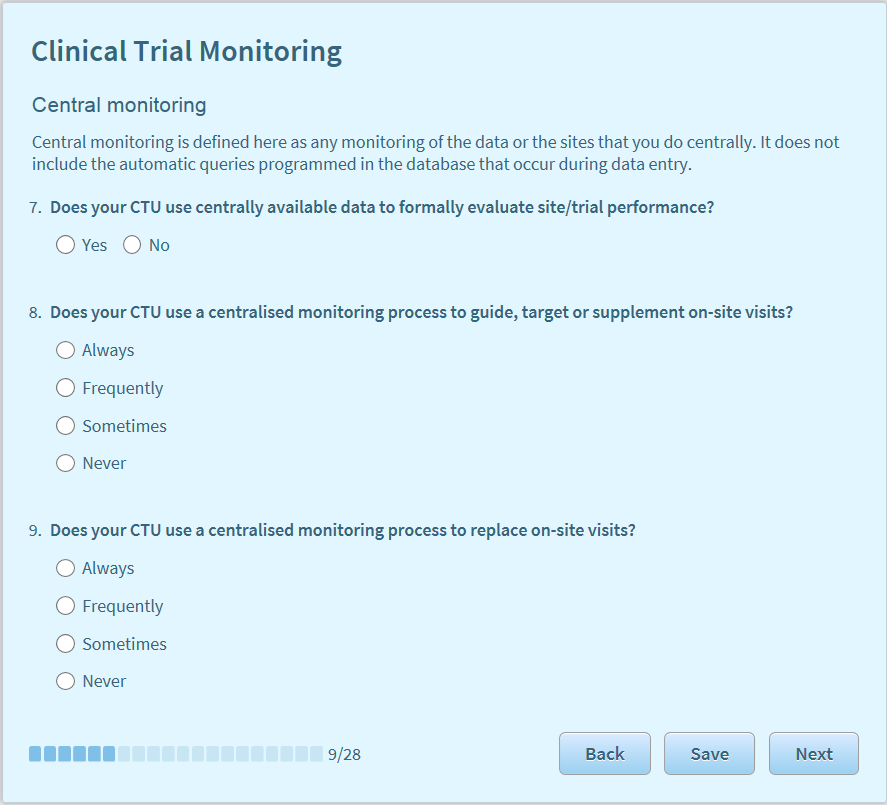


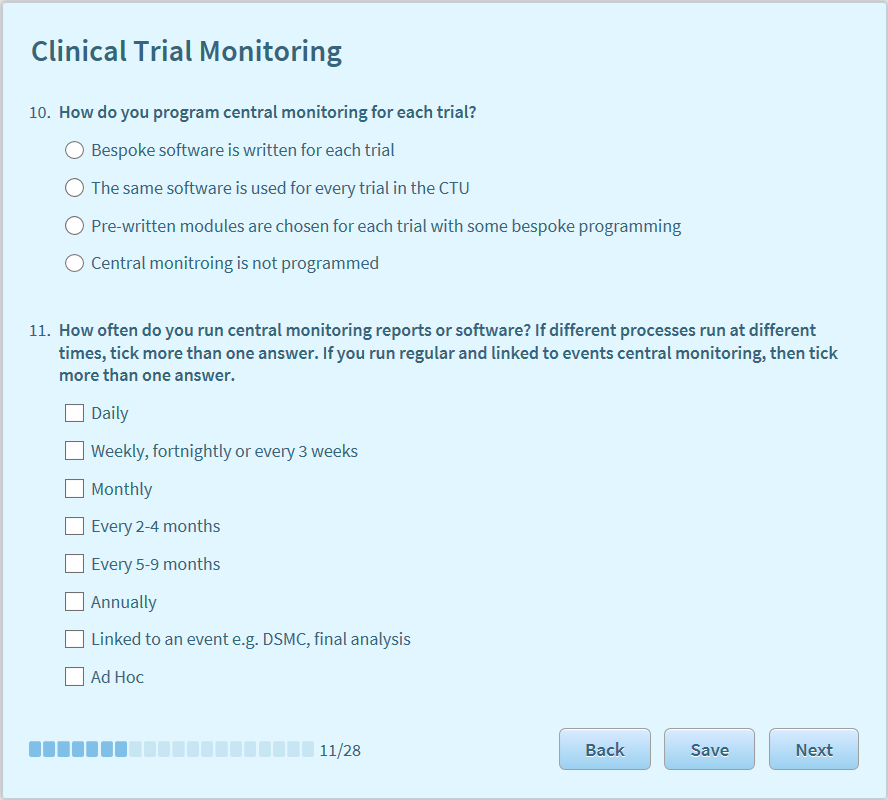


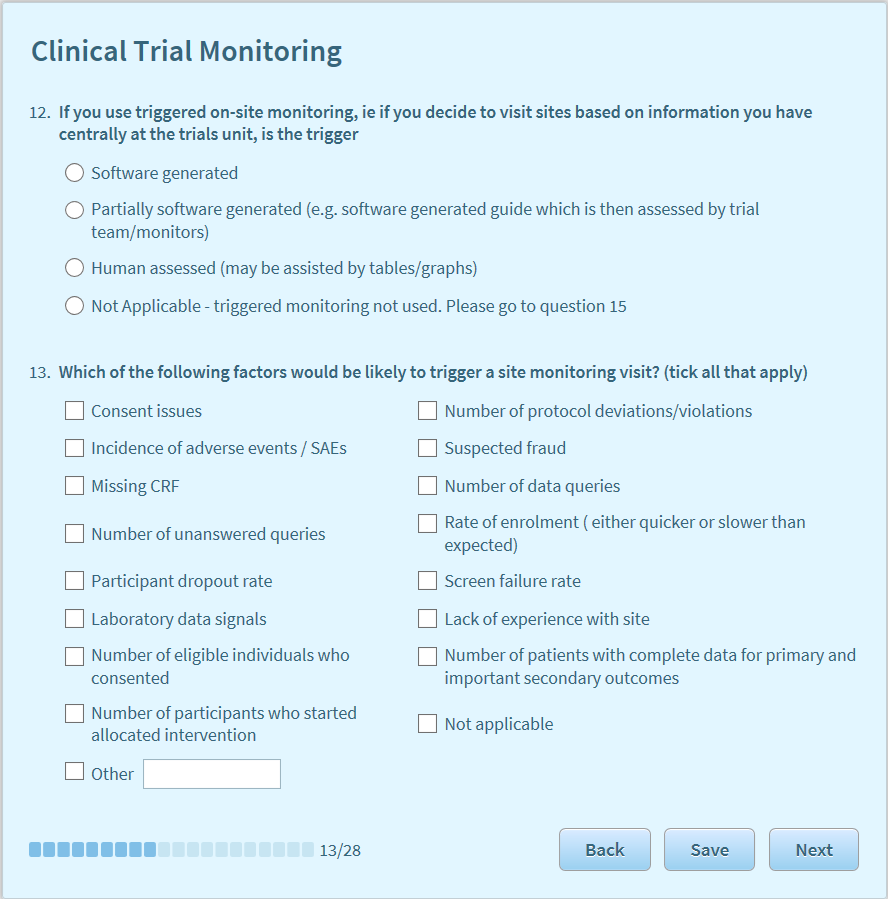


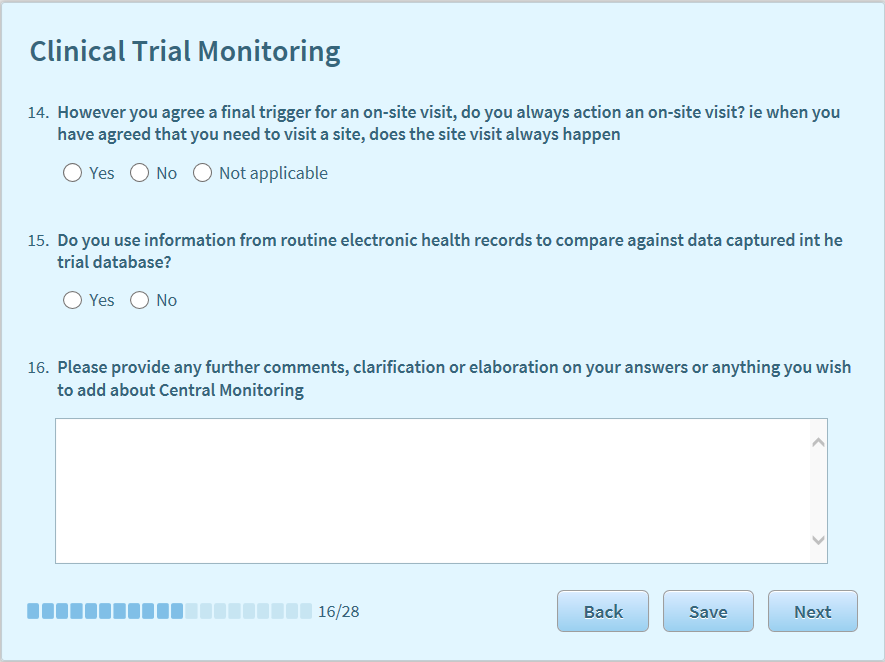


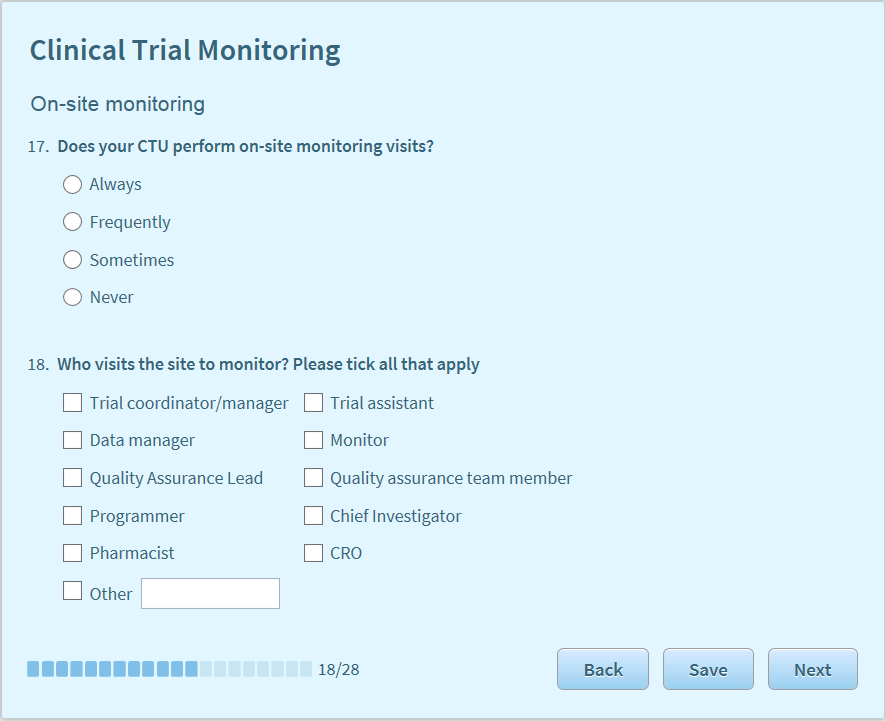


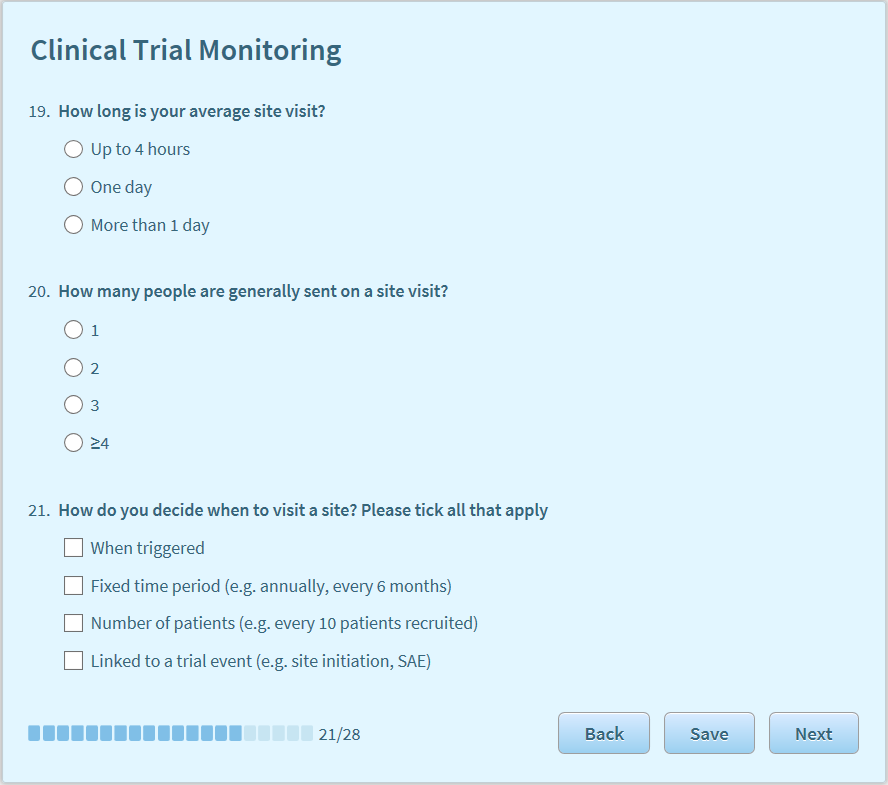


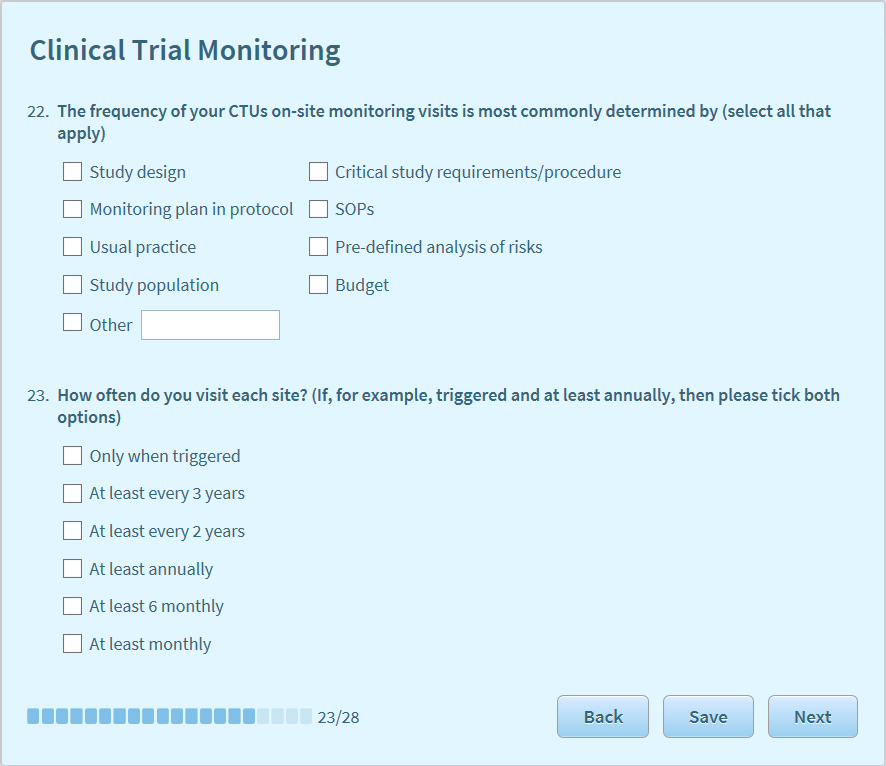


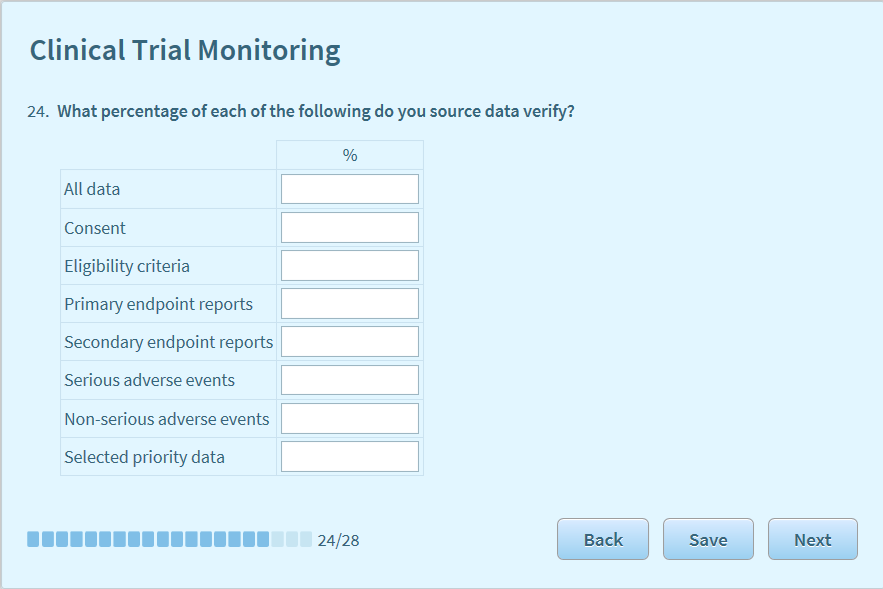


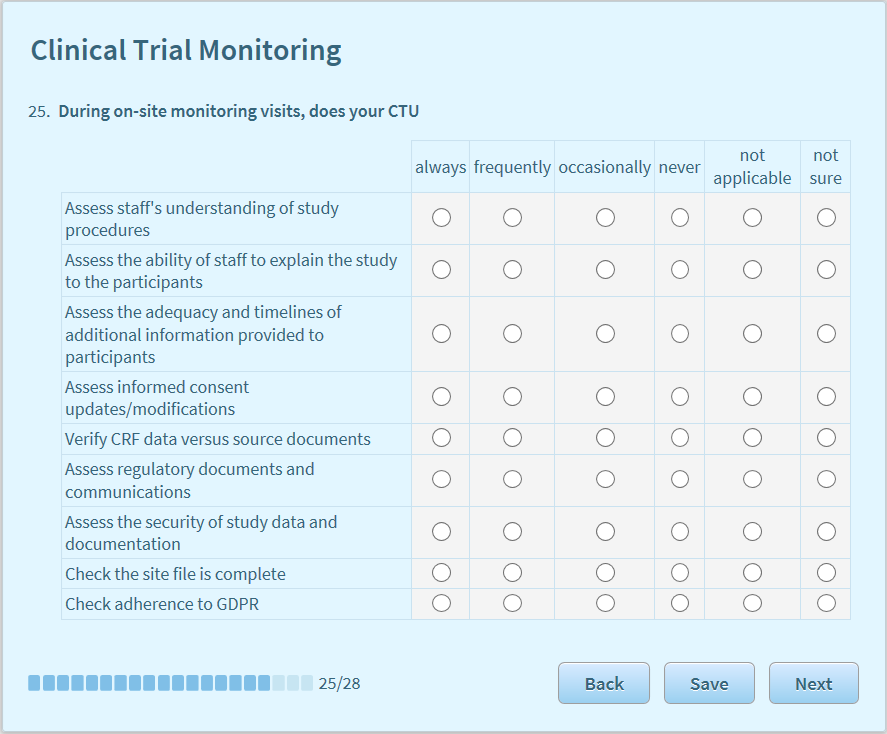


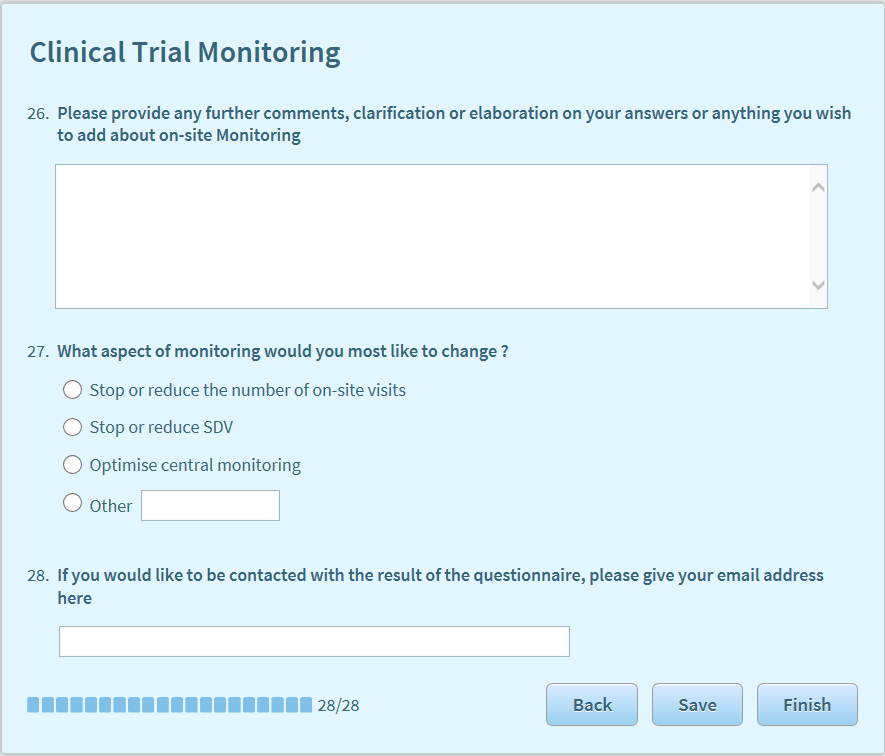


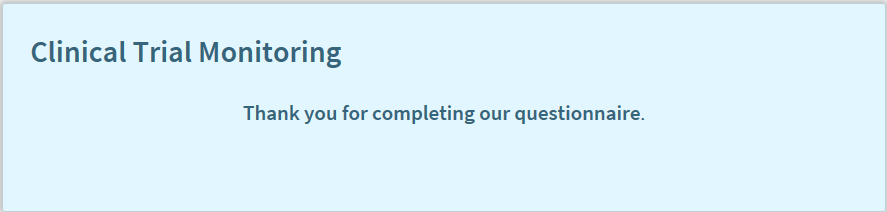

Supplement: Supplementary file 1 — Additional file 1. Screenshot CRCUK registered CTU questionnaire. [file 13063_2019_3976_MOESM1_ESM.docx]
