# Supplementary material for: Monitoring in practice – How are UK academic clinical trials monitored? A survey
Source: Trials. 2020 Jan 9;21:59. doi: 10.1186/s13063-019-3976-1 (PMC6953230; doi:10.1186/s13063-019-3976-1)
Supplement: Supplementary file 2 — Additional file 2. Transcript CRCUK registered CTU questionnaire. [file 13063_2019_3976_MOESM2_ESM.docx]

How do registered CTU in the UK monitor their trials?

| We are finding out how clinical trial monitoring is carried out in the UKCRC registered Clinical Trials Units. Please could you fill in the questionnaire thinking of the phase III randomised CTIMP trials led from your CTU. To avoid duplication, we have asked for your CTU name but we will not be using this for any analysis. The data will be stored in alignment with our CTU policy for trial data, it will only be accessible by those working on the project and will be analysed using STATA. We would like to publish the results of the questionnaire but will not be using the CTU names. For further information please contact [s.love@ucl.ac.uk](mailto:s.love@ucl.ac.uk) . For this questionnaire, monitoring is defined in the ICH way as  “The act of overseeing the progress of a clinical trial, and ensuring that it is conducted, recorded and reported in accordance with the protocol, Standard Operating Procedures (SOPs), Good Clinical Practice (GCP), and the applicable regulatory requirements”  “The purposes of trial monitoring are to verify that:  (a) The rights and well-being of human subjects are protected.  (b) The reported trial data are accurate, complete, and verifiable from source documents.  (c) The conduct of the trial is in compliance with the currently approved protocol/amendment(s), with GCP, and with the applicable regulatory requirement(s).”  <https://www.ich.org/fileadmin/Public_Web_Site/ICH_Products/Guidelines/Efficacy/E6/E6_R1_Guideline.pdf> (accessed 9Oct2018)  Thank you for your help |
| --- |

1. Trials at your CTU
2. Please enter your CTU name or registration number
3. What is the most common number of sites in your randomised phase III CTIMP trials?
   1. ≤10
   2. 11-49
   3. ≥50
4. What size randomised phase III CTIMP trials does your CTU usually conduct?
   1. ≤100
   2. 101-1000
   3. 1001-2499
   4. ≥2500
5. Does your CTU conduct international trials ?
   1. Yes
   2. No
6. Does your CTU conduct an assessment of risk to inform your monitoring approach?
7. Yes
8. Sometimes
9. No
10. Space for any comments on the questions above about randomised phase III CTIMP trials in your CTU

|  |
| --- |

1. Central monitoring

Central monitoring is defined here as any monitoring of the data or the sites that you do centrally. It does not include the automatic queries programmed in the database that occur during data entry.

1. Does your CTU use centrally available data to formally evaluate site/trial performance?
   1. Yes
   2. No
2. Does your CTU use a centralised monitoring process to guide, target or supplement on-site visits?
3. Always
4. Frequently
5. Sometimes
6. Never
7. Does your CTU use a centralised monitoring process to replace on-site visits?
   1. Always
   2. Frequently
   3. Sometimes
   4. Never
8. How do you program central monitoring for each trial?
9. Bespoke software is written for each trial
10. The same software is used for every trial in the CTU
11. Pre-written modules are chosen for each trial with some bespoke programming
12. Central monitoring is not programmed
13. How often do you run central monitoring reports or software? If different processes run at different times, tick more than one answer. If you run regularly and linked to events, then tick more than one answer.
14. Daily
15. Weekly, fortnightly or every 3 weeks
16. Monthly
17. Every 2-4 months
18. Every 5-9 months
19. Annually
20. Linked to an event e.g. DSMC, final analysis
21. Ad hoc
22. If you use triggered on-site monitoring ie if you decide to visit sites based on information you have centrally at the trials unit, is the trigger
23. Software generated
24. Partially software generated (e.g. software generated guide which is then assessed by trial team/monitors)
25. Human assessed (maybe assisted with tables/graphs)
26. Not Applicable - triggered monitoring not used. Please go to question 15
27. Which of the following factors would be likely to trigger a site monitoring visit? (tick all that apply)
28. Consent issues
29. Number of protocol deviations
30. Incidence of adverse events
31. Suspected fraud
32. Missing CRF
33. Number of data queries
34. Number of unanswered queries
35. Rate of enrolment (either quicker or slower than expected)
36. Participant dropout rate
37. Screen failure rate
38. Laboratory data signals
39. Lack of experience with site
40. Number of eligible individuals who consented
41. Number of patients with complete data for primary and important secondary outcomes
42. Number of patients who started allocated intervention
43. Not applicable
44. Other
45. However you agree a final trigger for an on-site visit, do you always action an on-site visit ? ie when you have agreed that you have a need to visit a site, does this site visit always happen?
46. Yes
47. No
48. Not applicable
49. Do you use information from routine electronic health records to compare against data captured in the trial database?
50. Yes
51. No
52. Please provide any further comments, clarifications or elaboration on your answer or anything you wish to add about Central Monitoring

|  |
| --- |

1. On site monitoring
2. Does your CTU perform on-site monitoring visits?
   1. Always
   2. Frequently
   3. Sometimes
   4. Never
3. Who visits the site to monitor? Please tick all that apply
4. Trial coordinator/manager
5. Trial assistant
6. Data manager
7. Monitor
8. Quality assurance lead
9. Quality assurance team member
10. Programmer
11. Chief Investigator
12. Pharmacist
13. CRO
14. Other
15. How long is your average site visit?
16. Up to 4 hours
17. One day
18. More than 1 day
19. How many people are generally sent on a site visit?
20. 1
21. 2
22. 3
23. ≥4
24. How do you decide when to visit a site (please tick all that apply)
25. When triggered
26. Fixed time period (e.g. annually, every 6 months)
27. Number of patients (e.g. every 10 patients recruited)
28. Linked to a trial event (e.g. site initiation, SAE)
29. The frequency of your trials on-site monitoring visits is most commonly determined by (select all that apply)
30. Study design
31. Critical study requirements/procedure
32. Monitoring plan in protocol
33. SOPs
34. Usual practice
35. Pre-defined analysis of risks
36. Study population
37. Budget
38. Other
39. How often do you visit each site? (If for example, it is triggered but at least annually, then please tick both options)
40. Only when triggered
41. At least every 3 years
42. At least very 2 years
43. At least annually
44. At least 6 monthly
45. At least monthly
46. What percentage of each of the following do you source data verify?

|  | % |
| --- | --- |
| All data |  |
| Consent |  |
| Eligibility criteria |  |
| Primary endpoint reports |  |
| Secondary endpoint reports |  |
| SAE – Serious adverse events |  |
| AE – Non-serious adverse event reports |  |
| Selected priority data |  |

1. During on-site monitoring visits, do you

|  | always | frequently | occasionally | never | Not applicable | Not sure |
| --- | --- | --- | --- | --- | --- | --- |
| assess staff’s understanding of study procedures |  |  |  |  |  |  |
| assess the ability of staff to explain study to participants |  |  |  |  |  |  |
| assess the adequacy and timelines of additional information provided to participants |  |  |  |  |  |  |
| assess informed consent updates/modifications |  |  |  |  |  |  |
| verify CRF data versus source documents |  |  |  |  |  |  |
| assess regulatory documents and communications |  |  |  |  |  |  |
| assess the security of study data and documentation |  |  |  |  |  |  |
| Check the site file is complete |  |  |  |  |  |  |
| Check adherence to GDPR |  |  |  |  |  |  |

1. Please provide any further comments, clarification or elaboration on your answers or anything you wish to add about on-site monitoring

|  |
| --- |

1. Which aspect of monitoring would you most like to change?
   1. Stop or reduce the number of on-site visits
   2. Stop or reduce SDV
   3. Optimise central monitoring
   4. Other

Thank you for completing our questionnaire
