# Supplementary material for: Monitoring in practice – How are UK academic clinical trials monitored? A survey
Source: Trials. 2020 Jan 9;21:59. doi: 10.1186/s13063-019-3976-1 (PMC6953230; doi:10.1186/s13063-019-3976-1)
Supplement: Supplementary file 3 — Additional file 3. Frequency of differing combinations of people attending on on-site monitoring visits. [file 13063_2019_3976_MOESM3_ESM.docx]

Additional file 3 – Frequency of differing combinations of people attending on on-site monitoring visits

| Fre-quency  N=34 | Trial Coordinator/ Manager | Trial Assist-  ant | Data Man-ager | Moni-tor | Quality Assurance Lead | Quality Assurance Team Member | Program-mer | Chief Investigator | Pharm-acist | Contract Research Organisation | Other |
| --- | --- | --- | --- | --- | --- | --- | --- | --- | --- | --- | --- |
| 7 | y |  |  |  |  |  |  |  |  |  |  |
| 6 | y |  |  | y |  |  |  |  |  |  |  |
| 3 |  |  |  | y |  |  |  |  |  |  |  |
| 2 | y |  |  | y | y | y |  |  |  |  |  |
| 2 | y |  | y |  |  |  |  |  |  |  |  |
| 1 | y | y |  |  |  |  |  |  |  |  |  |
| 1 | y | y |  |  | y |  |  |  |  |  |  |
| 1 | y |  | y | y |  | y |  |  |  |  |  |
| 1 | y |  | y |  |  |  |  | y |  |  |  |
| 1 |  |  |  | y | y |  |  |  |  | y |  |
| 1 |  |  |  | y |  | y |  |  |  |  |  |
| 1 | y |  | y | y |  |  |  |  |  | y |  |
| 1 |  |  |  | y | y | y |  |  |  |  |  |
| 1 | y |  |  |  |  | y |  |  |  |  |  |
| 1 | y | y |  | y |  |  |  |  |  |  |  |
| 1 | y |  | y |  |  |  |  |  |  |  | * |
| 1 | y |  |  | y | y | y |  | y |  |  |  |
| 1 | y |  |  | y |  |  |  |  |  | y |  |
| 1 | y |  | y |  |  |  |  |  |  |  | # |
|  |  |  |  |  |  |  |  |  |  |  |  |
|  |  |  |  |  |  |  |  |  |  |  |  |
| * Local monitor # Clinical Project Manager (including the CPM for monitoring) as required | | | | | | | | | | |  |
